# Evaluating the Effects of an Algebraic Frequency Building Intervention for Students with Disabilities

**DOI:** 10.1007/s40617-025-01082-9

**Published:** 2025-07-31

**Authors:** Olivia G. Enders, Douglas Kostewicz

**Affiliations:** 1 Department of Teacher Education Spadoni College of Education and Social Sciences, Coastal Carolina University, 100 Chanticleer Dr E, Prince Building, 100 Tom Trout Dr E, Conway, SC 29528 USA; 2 Department of Teaching, Learning, & Leading; School of Education, University of Pittsburgh, 5500 Wesley W. Posvar Hall, Pittsburgh, PA 15260 United States of America

**Keywords:** Math intervention, Precision teaching, Learning disabilities, Equations

## Abstract

Evidence-based instructional strategies in mathematics provide critical support related to academic achievement for students with disabilities. Many researched mathematics interventions focus on foundational and elementary mathematics skills, indicating the need for interventions for higher-level topics such as algebra. Current research on a core skill within algebra—solving equations—focuses primarily on the initial acquisition. To move students past acquisition, we must consider how interventions target behavioral fluency. Leveraging precision teaching practices, we evaluated a frequency-building intervention to improve the rates of three middle-school students’ correct responses to one-step algebraic equations using a multiple-baseline across participants design. Results demonstrate immediate and significant positive effects on students’ correct notations and drastic decreases in students’ incorrect notations. Students generalized skills to untaught problem presentations. In pre- and post-think-aloud, students moved from using trial-and-error methods to using systematic procedures to solve equations. We discuss how the characteristics of the intervention supported students’ overall improvement and discuss potential implications for both practice and future research.

• Frequency-building interventions are effective in supporting students’ accuracy and speed in solving algebraic equations

• Precision teaching, including the use of the Standard Celeration Chart, is a powerful system for guiding both research and instructional decisions

• Fluent behavior in foundational skills can support generalization to new mathematical problems

• Mathematics research broadly must incorporate timing to facilitate movement beyond the acquisition stage of learning

Proficiency in algebra increases educational opportunities for students (Gaertner et al., 2014; Kilpatrick & Iszák, 2008; Morgatto, 2008). Pre-algebraic skills provide students with basic tools to attain more complex quantitative skills presented in algebra I and II and calculus (Usiskin, 1995). Students enrolled in algebra and subsequent classes are more likely to graduate from high school, enter college, and maintain higher grade point averages in college than those who do not take advanced math courses (Gaertner et al., 2014; Kim et al., 2015). Additionally, as science, technology, engineering, and mathematics, or STEM, jobs in the United States continue to grow, a repertoire of complex math skills can provide opportunities to obtain higher-wage, readily available jobs (Fayer et al., 2017).

Secondary students with disabilities have historically struggled to attain algebraic proficiency on standardized assessments. Data from the 2022 National Assessment of Educational Progress Report Card in Mathematics suggest eighth-grade students with disabilities continue to underperform on tests of achievement in comparison to their non-disabled peers and have reduced odds of entering algebra in eighth grade (Nation’s Report Card, n.d.; Faulkner et al., 2013). Given the importance of algebraic proficiency, identifying evidence-based practices in mathematics remains a critical endeavor. Research indicates students with disabilities may struggle with the prerequisite skills required for success in algebra, including early numeracy skills, fraction knowledge, and computational fluency (Jordan et al., 2017; Nelson & Powell, 2018). Interventions subsequently target both pre-requisite skills and evidence-based strategies for introducing algebraic skills.

Researched interventions specifically targeting algebra encompass multiple promising practices for students with learning disabilities (Bone et al., 2021; Lee & Mao, 2020;) General strategies for algebraic skills include the use of heuristics, the use of manipulatives, schema-based instruction, peer-assisted learning strategies, student verbalizations, concrete-representational-abstract (CRA) frameworks, and explicit instruction (Bone et al., 2021; Lee & Mao, 2020). Researchers often evaluate multiple strategies within an intervention package. For example, a study may focus on the impacts of explicit instruction paired with CRA frameworks (Bone et al., 2021).

Across the literature, researchers often evaluated intervention efficacy via accuracy (i.e., percentage correct) rather than via behavioral fluency (i.e., speed and accuracy; Binder, 1996). A focus on accuracy indicates interventions often target the initial acquisition of a skill. However, mathematics often necessitates students combine element skills within a more complex task. Element skills are discrete tasks, such as solving basic multiplication facts (Stocker et al., 2019). Compound skills, such as solving a long division problem, necessitate the execution of multiple-element skills. Completing element skills quickly and accurately may be valuable in preventing cumulative dysfluency when students cannot efficiently complete compound skills within complex tasks (Evans et al., 2021; McDowell & Keenan 2001).

Element skills in mathematics may include simple computations. They may also describe discrete steps in a larger task, such as executing the order of operations when simplifying an expression. As students gain fluent responding in element skills, individuals can execute compound skills with greater accuracy. Furthermore, fluent responding is associated with various critical learning outcomes (Binder, 1996; Kubina & Yurich, 2012; McTiernan et al., 2016; Stocker et al., 2019). Critical learning outcomes include retention, endurance, application to novel or more complex activities, maintenance over time, and stability. Thus, ensuring fluent responding on element skills can support the acquisition of more complex skills and support students’ generalization and application of skills.

To target fluent behavior, researchers may consider the use of precision teaching. As defined by Evans et al. (2021), precision teaching is “a system for precisely defining and continuously measuring dimensional features of behavior and analyzing behavioral data on the [Standard Celeration Chart] to make timely and effective data-based decisions to accelerate behavioral repertoires” (p. 561). Within precision teaching, researchers often use a practice called frequency-building, or systematic practice, to support the acceleration of behaviors (Gist & Bulla, 2020; Kubina & Yurich, 2012). Frequency-building refers to timed practice sessions in which a student repeatedly practices a specific behavior, working toward a performance standard indicative of fluent responding (Gist & Bulla, 2020; Kubina & Yurich, 2012). To support accurate responding, an interventionist may deliver performance feedback (Gist & Bulla, 2020; Kubina & Yurich, 2012).

A systematic review of frequency-building interventions identified only four studies in the domain of mathematics; all four studies focused on simple computations not exceeding multiplication (Gist & Bulla, 2020). An additional review focusing broadly on behavioral fluency and mathematics also found no studies targeting algebra (Stocker et al., 2019). More recently, one study examined the effects of a self-managed intervention on students’ behavioral fluency related to pre-algebraic tasks (Stocker & Kubina, 2021). Both reviews, as well as the most recent study, found improved student performance because of interventions targeting behavioral fluency.

The current study moves our focus beyond the initial acquisition of algebraic skills for students. We use precision teaching and frequency-building to target students’ behavioral fluency in a critical foundational skill in mathematics—solving equations. Employing a multiple-baseline, multiple-probe across participants design, we sought to interrogate the effects of a frequency-building intervention on students with learning disabilities’ rate of correct and incorrect notations while solving one-step equations.

## Methods

### Participants and Setting

Alyssa, Jacob, and Kyleigh, the three participants in the study, attended a public charter school specializing in serving students with learning disabilities in a midsize Midwestern city. The school includes grades two through eight and enrolls students based on a lottery system. In 2021, 310 students were enrolled with a 6:1 student-to-teacher ratio. The majority (64.84%) of students were white, with fewer Black (25.25%), multiracial (7.98%), Hispanic (2.33%), and Asian (< 1%) students. The school received Title I funding.

Alyssa was a 12-year-old Black female in the sixth grade. Alyssa qualified for special education services via a specific learning disability (SLD) in mathematics. Alyssa received four days of intensive mathematics intervention per six-day class rotation at the school. Jacob was a 12-year-old White male in the sixth grade. Jacob qualified for special education services via a diagnosis of SLD in reading and mathematics. Jacob received four days of intensive mathematics intervention per six-day class rotation at the school. Kyleigh was a 13-year-old White female in the seventh grade. Kyleigh qualified for special education services via a diagnosis of SLD in reading and mathematics. Kyleigh received two days of intensive mathematics intervention per six-day class rotation at the school. Owing to data sharing limitations, no other assessment data related to the students’ academic and cognitive performance was provided.

The lead author served as the interventionist for the study. At the time of intervention, the lead author was a doctoral student in a PhD program specializing in Special Education and had previously worked as a learning support teacher in middle school mathematics classrooms. The interventionist conducted sessions 1:1 with students in a separate office designated for intervention. Sessions were conducted daily, Monday through Friday, when possible. Absences, weather cancellations, and other school functions, like assemblies and school-wide meetings, sometimes prevented daily implementation. The study spanned 54 calendar days.

### Materials

#### Dependent Variable Assessment Sheet

**Fig. 1:**
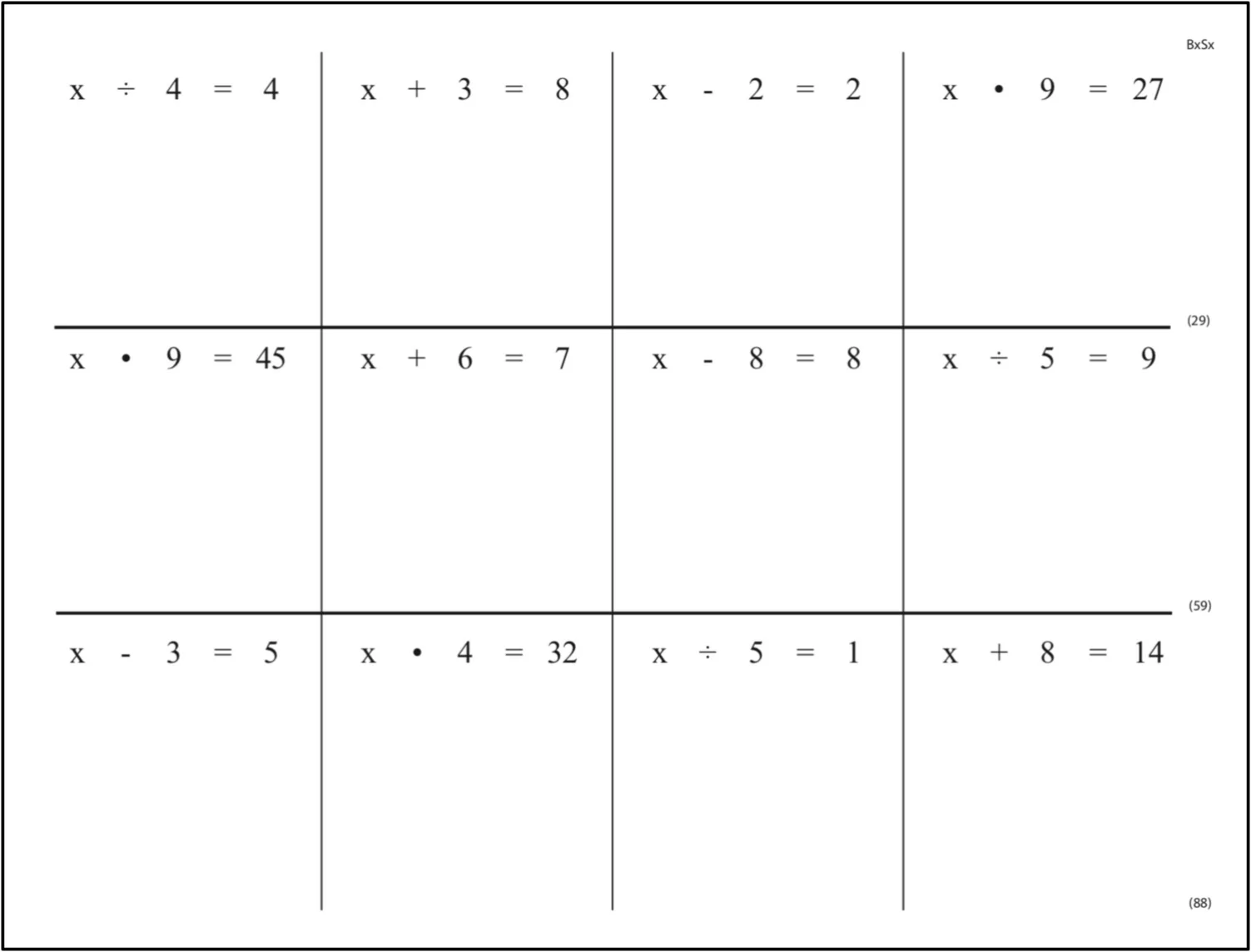
Dependent variable assessment sheet

We created materials for screening and frequency-building sessions based on the implementation guidelines in The Precision Teaching Implementation Manual (Kubina, 2019) and in conjunction with experts in precision teaching. The first author created dependent variable assessment sheets by listing all basic math facts and creating a one-step equation that related to each fact. We then distributed the problems across four bins of problems. When creating a probe sheet, each sheet was created from a single bin of problems to ensure any given probe would not include a disproportionate number of equations with a specific number. For example, problems requiring multiplication or division with the number 1 were distributed evenly across bins. We also evenly distributed problem difficulty across bins and probes sheets. For example, one sheet would not contain two problems where the student only had to add 1 to each side. Then, we created sheets using approximately the same number of one-step addition, subtraction, multiplication, and division problems on each page for a total of 16 unique sheets. An individual fluent in one-step equations completed each sheet to verify equivalent difficulty, as measured by time to completion. Each sheet contained nine one-step equations; each one-step equation required at least seven correct notations to complete. Sheets were used randomly across baseline and intervention to ensure equivalent difficulty. Figure 1 depicts the dependent variable assessment sheet.

#### Instructional and Think-Aloud Materials

**Fig. 2:**
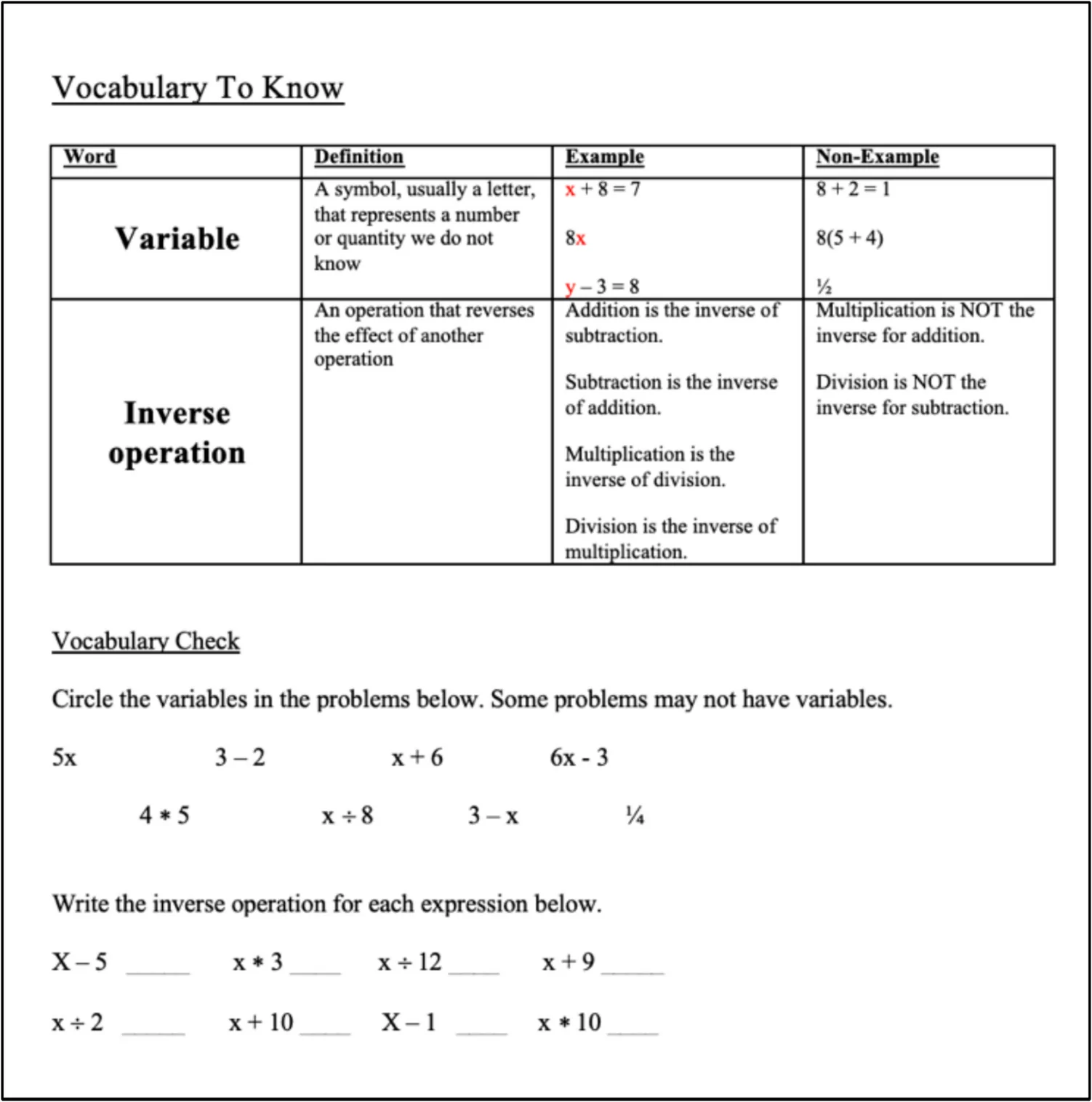
Initial vocabulary instruction sheet

Instructional and think-aloud materials consisted of two whiteboards, two markers, a voice recorder, a stopwatch, and practice sheets. The initial instructional sheet included vocabulary definitions, examples, and non-examples of two terms, inverse operations and variables, and is displayed in Fig. 2.

**Fig. 3:**
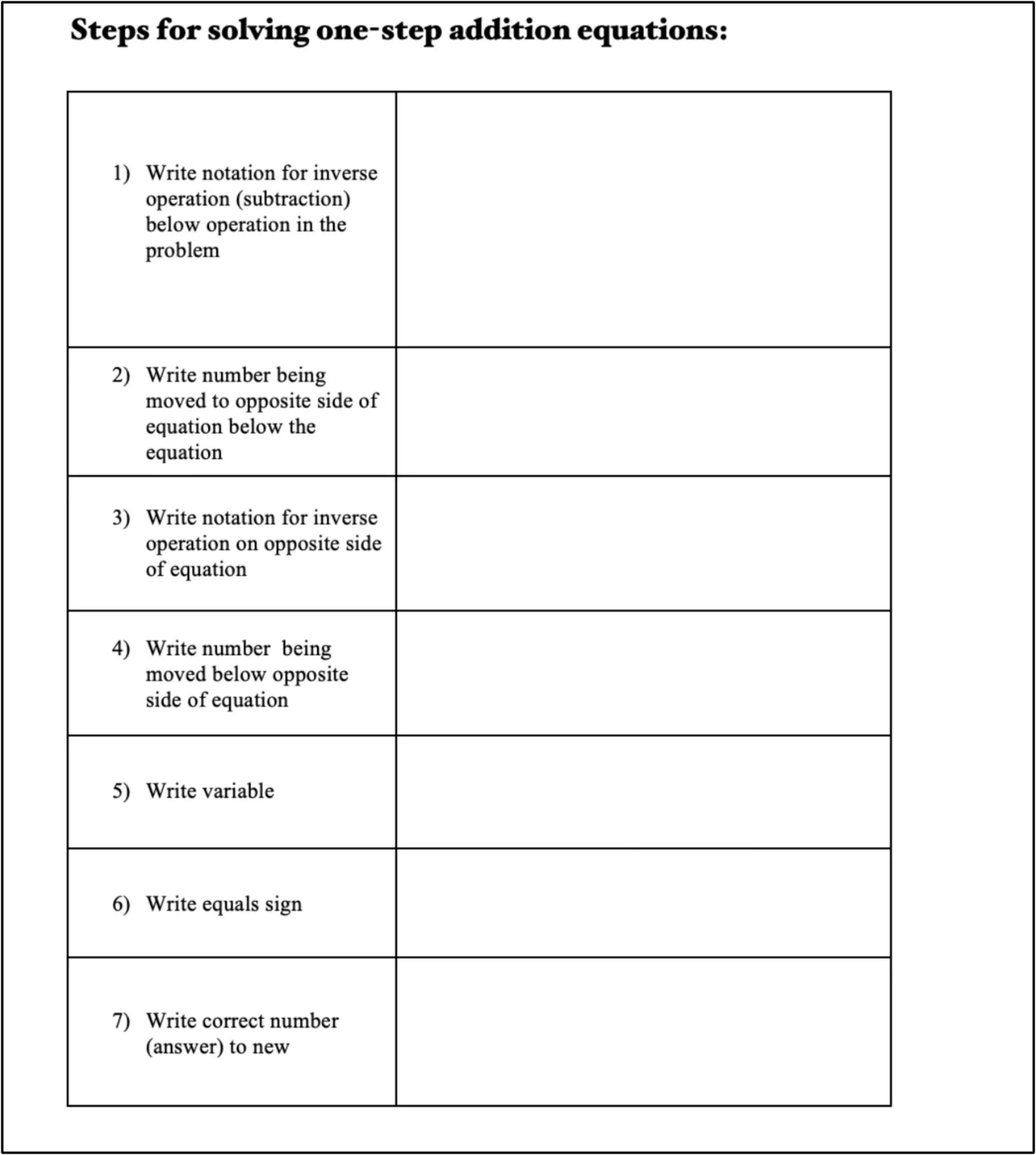
Guided practice sheet

Four guided practice sheets were used for instruction of the task analysis, with one practice sheet per type of problem (e.g., one-step addition, one-step subtraction). The back of the sheet included eight independent practice problems. Figure 3 depicts guided practice sheets. Baseline and intervention materials included a stopwatch, pencil, and intervention script, as well as the dependent variable assessment sheets and frequency-building sheets.

## Primary and Secondary Dependent Variables

**Fig. 4:**
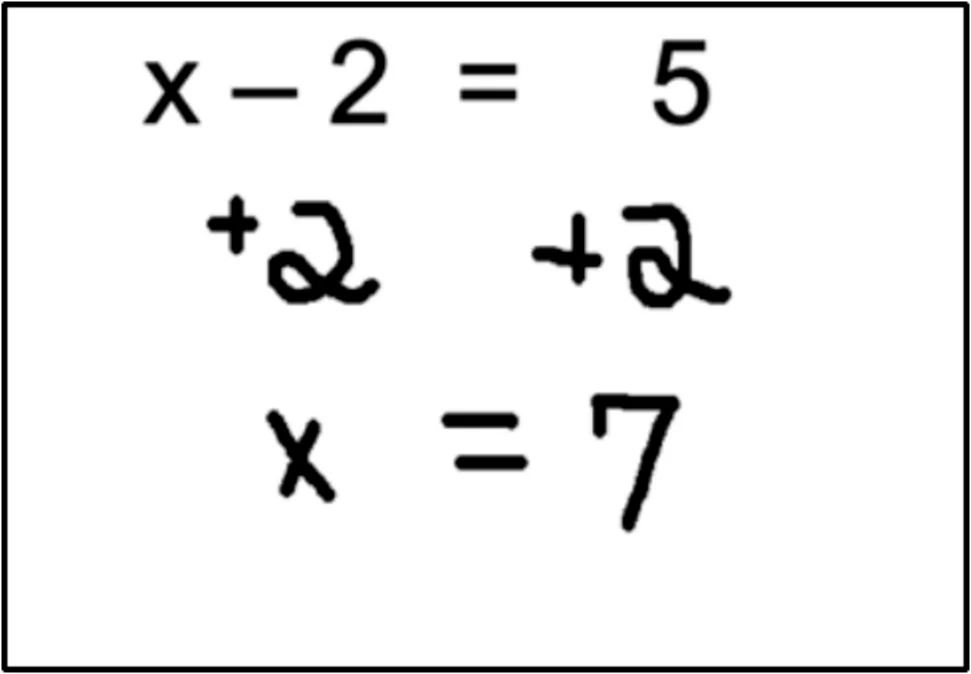
Example of correct notations

The primary dependent variable included correct and incorrect notations per minute. A correct response included any legible notation written in the correct location. We defined correct location as notations that followed steps in the task analysis for solving one-step equations. Operation signs and numbers counted discretely; for example, writing −6 underneath each side of the equation “*x* + 6 = 10” counted as four correct notations: two for each minus sign and two for each 6. In total, each problem had a minimum of seven opportunities for correct notations. Students could write numbers backward but had to include the correct number—for example, after subtracting “10 − 6,” students had to write the number “4” for the response to be marked correct; writing the number “5” was labeled as an incorrect response. Additionally, numbers or notations written in the incorrect location, such as on the wrong side of the equation, were marked as incorrect. Partial problems were counted, such as responses leading up to the correct answer but not having the final correct answer, were counted as correct or incorrect. The researcher did not count skipped problems or problems with no notations as incorrect or correct notations. Each correct notation corresponded to a step of the task analysis described below. Figure 4 displays a problem with seven correct notations underneath a problem.

**Fig. 5:**
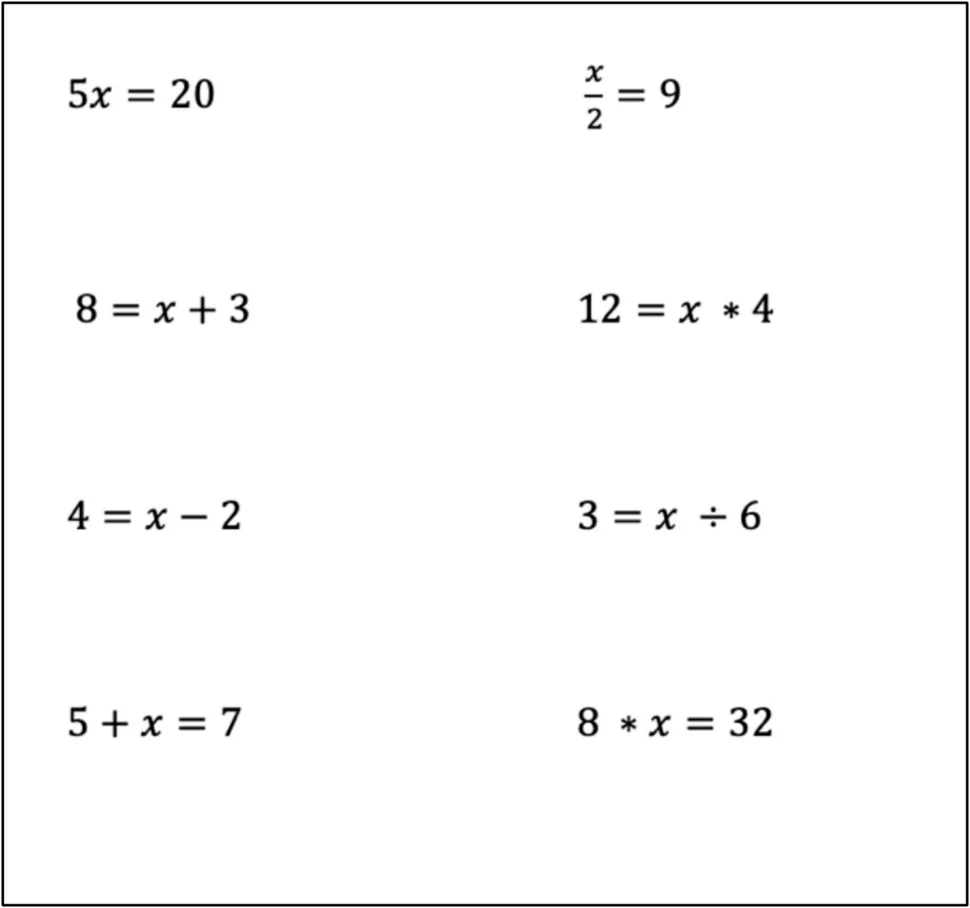
Generalization probe

We recorded student verbalizations in the think-aloud activities to analyze as a secondary variable. The lead author transcribed student verbalizations from the recordings and compared changes across pre- and post-think-aloud activities in response to the same equation. We also evaluated students’ generalization of skills to varied presentations of problems using the generalization sheets. The sheets contained eight problems with non-taught presentations of one-step equations. Presentations included changing the order of the variable (e.g., 6 + *x* = 5) or presenting operations using different symbols (e.g., fraction bars). Generalization probes did not require students to complete any additional steps, as modifications related only to the presentation of problems. Figure 5 displays the generalization probe.

### Procedures

#### Screening

We identified eligible students by working with school personnel to identify students with a diagnosed disability via an individualized education plan (IEP). Students had to have a math goal indicating math as an area of need and had to participate in a math intervention block. After meeting the initial criteria, the lead author disseminated IRB-approved informational letters and consent forms to the parents or caregivers of the students describing the study’s goals. We attained consent from parents and assent from students via conversation, which included a description of procedures in the study and active affirmation of student participation. The lead author then screened students to ensure the presence of prerequisite skills necessary for participation.

The screening included students completing a timed, 30-second probe of randomly distributed single-digit addition, subtraction, multiplication, and division problems (i.e., the basic 390 math facts; one type of operation per sheet). All participants fell in the middle 50th percentile range, indicating they were not at the frustration level of instruction on basic computational skills. Students at the frustration level with computational skills would not have benefitted from a focus on compound skills. The second section of screening assessments contained 12 one-step equations and was identical to the dependent variable assessment sheet and the sheets used during frequency building, described below. We developed the performance standard of 60–80 correct notations per minute by sampling fluent learners on the screening assessment (Kubina & Yurich, 2012). No student exceeded 13 correct notations on the screener, suggesting they would benefit from frequency-based instruction.

#### Independent Variable Procedures

The independent variable consisted of one day of instruction, followed by a daily frequency-building intervention. The one day of instruction included explicit instruction of the vocabulary terms inverse operation and variable. Then, we modeled steps of solving one-step equations, which was followed by guided and independent practice. For modeling and guided practice, we used the following task analysis, with each step corresponding to a notation:

1. Write notation for inverse operation below operation in the problem.
2. Write number being moved to opposite side of equation below the equation.
3. Write notation for inverse operation on opposite side of equation.
4. Write number being moved below opposite side of equation.
5. Write variable.
6. Write equal sign.
7. Write correct number opposite equal sign.

The instructional session began with a stated objective: to solve one-step equations accurately by finding the value of the unknown or the variable. The lead author provided vocabulary instruction on the terms “variable” and “inverse operation.” Students had to identify examples of the variable and inversed operations in context in five consecutive trials with 100% accuracy before moving on to the next instructional phase. Next, the interventionist reviewed the seven-step task analysis using modeling and verbal repetition of the steps of the task analysis, with students completing the identical problems on a whiteboard. Then, students moved to guided practice on problems using a least-to-most system of prompting and the displayed written task analysis. After completing the guided practice for a single operation, students completed eight independent practice problems. Students had to complete at least seven of eight problems correctly to exit the instruction phase for a given operation. The process was repeated across each operation. No instructional session lasted longer than one day for the three participants.

After the day of the instructional session, students moved to the frequency-building intervention. Daily frequency-building sessions began with a dependent variable assessment, followed by three 1-minute sessions of practice and feedback. The dependent variable assessment was identical to the baseline assessment. The interventionist reminded each student to work as quickly as possible and to work from left to right. After the student completed the dependent variable assessment, the interventionist provided positive statements about the student’s effort in completing the assessment.

Next, students engaged in three 1-minute frequency-building sessions, each followed immediately by the provision of feedback. At the beginning of each 1-minute session, the first author stated the objective of the frequency-building sessions and provided their prior correct number of notations in the dependent variable assessment. The instructor explained to the student that they would be working as fast as they could to advance past their prior correct notations and started the timer. After each student had worked for one minute, they prompted them to stop working and provided feedback. If students made a computational error, the lead author provided feedback using a model–lead–test format for error correction. If the student made an error related to the task analysis, the lead author identified the error by connecting the error to the step of the task analysis. For example, if students added instead of subtracting when isolating the variable, the researcher pointed to the error and stated to use the inverse operation (i.e., subtraction). The length of feedback varied depending on the number of student errors. The total intervention length lasted between approximately seven and ten minutes.

#### Treatment Fidelity Procedures

We created a checklist with explicit steps that corresponded to each session. The checklist was visible and present for all sessions. We also audio-recorded 25% of the data collected in baseline and intervention and scored procedural fidelity via a trained observer. We calculated procedural fidelity for a single session by dividing the number of steps observed by the total steps possible and multiplying by 100. In addition to identifying procedural fidelity, the lead author took notes about any special or extenuating circumstances (e.g., student illness, student fatigue). All intervention sessions demonstrated 100% fidelity. One baseline session did not include the direction for students to work from left to right for 80% fidelity, although the student performance did not change from previous baseline sessions. All other baseline sessions were completed with 100% procedural fidelity. Recorded intervention sessions lasted an average of 8 min, 17 s.

## Experimental Design and Data Analysis

We used a multiple probe, multiple baseline across participants design to evaluate the effects of the frequency-building intervention on correct and incorrect notations. We began baseline probes with all students at the start of the study. We collected baseline data on each student for five days. Consistent with Precision Teaching practices, we calculated metrics associated with the Standard Celeration Chart (SCC) to analyze and guide our research design (Kubina, 2019). After each student’s fifth day, we derived the improvement index (II) to guide decision-making on the order of entry. II describes the overall improvement or betterment of the dependent variable (Kubina, 2019). The student with the lowest decelerating II entered first, and subsequent entries into the intervention followed this rule. We collected data on students who entered the intervention for a minimum of five days before having the next student enter. Students not in instruction remained in baseline. We collected baseline probes for students every third school day, as possible. Students needed to have an II of × 1.25 (or a 25% increase) within the frequency-building intervention for the next student to receive instruction and then intervention. If the student hit the performance standard and had a minimum of five data points, we exited students from the intervention phase. Otherwise, students remained in the study for at least 10 intervention days, a limit imposed by time constraints in the school setting.

We graphed the primary dependent variable on the SCC and derived statistics using the SCC to conduct data analysis. The SCC uses standardized notations and displays to support objective quantification of data beyond the scope of visual analysis (Kubina, 2019). Analysis occurred both within and between conditions, so long as sufficient data points existed to adequately represent the pattern ofs behavior (Kubina, 2019). Within conditions refers to the analysis of data within either the baseline or intervention phase, while between conditions compares values derived from student performance in baseline and intervention.

Within conditions, we considered the level, celeration, and II values. We calculated level by finding the geometric mean due to statistical advantages (Clark-Carter, 2005). Level calculations communicated the average level of responding in a phase. Celeration values represented the change in frequency of a behavior in time divided by time or how fast the behavior changed (Johnston & Pennypacker, 2009; Kennedy, 2005; Kubina, 2019). Celeration encompasses both acceleration (increasing frequency across time, represented with an × symbol) and deceleration (decreasing frequency, represented with a ÷ symbol) of correct and incorrect notations. To calculate II within a condition, we compared the celeration of incorrect and correct notations. If both incorrect and correct notation celerations had the same signs, we divided the larger value by the smaller; if the signs were different, we multiplied. We assigned the sign based on how the overall performance was changing. Such metrics were critical given the focus of this study on increased speed and accuracy.

Between conditions, we compared frequency multiplier and level, celeration, and II change. The frequency multiplier quantifies the immediacy of change between any two frequencies (Kubina, 2019). Specifically, we examined the final frequency of baseline to the first frequency of intervention. We compared level change between conditions by dividing the larger value by the smaller value and using the sign that represents the change (× for increase, ÷ for decrease). Level change captured differences in geometric means between conditions. A meaningful change was ×2 or greater (Kubina, 2019). We calculated the celeration multiplier to compare celeration between conditions. The celeration multiplier represented the speed change between conditions. We calculated the celeration multiplier to compare conditions using the same formula described for the II. Similarly, we used the same formula to calculate changes between conditions within the II. Comparison of the II accounts for correct and incorrect notations and produced the II change (Kubina, 2019).

We measured social validity via a Likert scale survey, which we delivered to students participating in the intervention condition. Per Wolf (1978) and Horner et al. (2005), social validity questions asked, in audience-appropriate language, whether the outcomes were socially important, whether the magnitude of change was important, whether implementation was practical and time- and cost-effective, and whether the intervention was likely to continue after the research project was finished. The scale was experimenter created. Additional open-ended questions targeted what parts of the intervention were acceptable to students and what parts were not. Open-ended questions were also experimenter created. The researcher provided students with both open- and closed-ended questions one week after each student finished intervention.

## Results

**Fig. 6:**
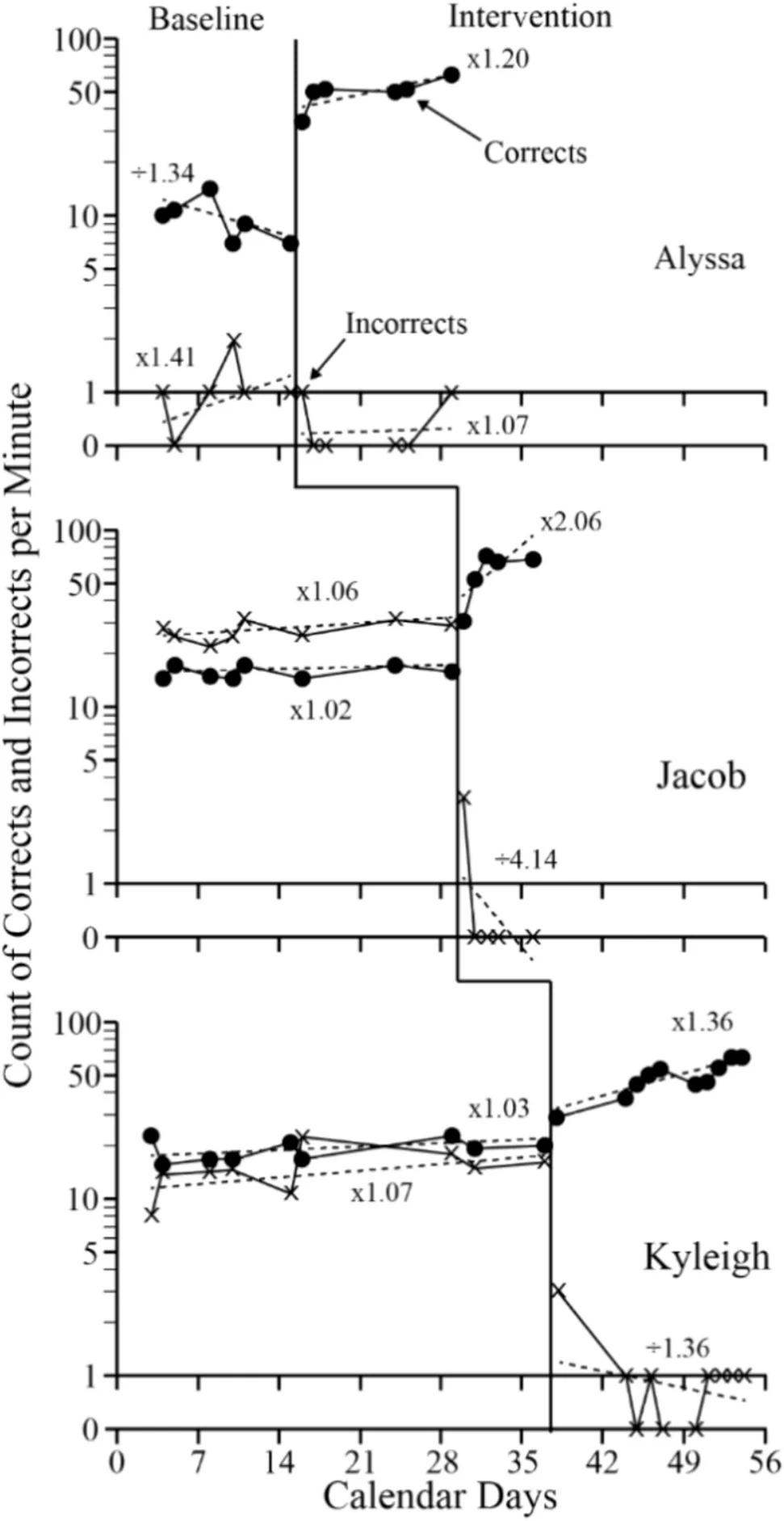
Standard celeration chart displaying correct and incorrect notations across participant

Figure 6 is an SCC illustrating correct and incorrect notations per minute on dependent variable assessments across baseline and intervention for Alyssa, Jacob, and Kyleigh. Dots correspond to correct notations, and incorrect notations are noted with x’s. The vertical axes refer to the number of notations per minute on a ratio scale. The x axes represent calendar days scaled linearly. Dotted lines represent celeration lines derived from a linear regression formula.

Table 1 depicts the level (geometric mean), celeration, bounce, and II in baseline and intervention conditions for Alyssa, Jacob, and Kylie. In baseline, Alyssa and Kyleigh both had higher levels of correct notations than incorrect notations, with Jacob displaying the inverse. Jacob and Kylie had accelerating correct and incorrect notations per minute, while Alyssa had decelerating corrects (÷ 1.34). Bounce scores were similar for both correct and incorrect notations for Alyssa and Jacob. Kyleigh displayed greater bounce for incorrect notations (× 2.30) rather than correct notations. As an overall picture, all three students had declining II scores in baseline, meaning that the overall quality of their performance was deteriorating across the condition.

**Table 1:** Within-condition measures of student notations

			Baseline						Intervention			
Student	NT	Level	*SD* (× /÷)		Cel.	Bounce	II	Level	*SD* (×/÷)	Cel.	Bounce	II
Alyssa	C I	9.38 0.70		1.30 0.55	÷ 1.34 × 1.14	× 1.80 × 2.00	÷ 1.89	47.13 0.26	1.21 0.43	× 1.20 × 1.07	× 1.40 × 2.00	× 1.12
Jacob	C I	15.78 26.67		1.13 1.14	× 1.02 × 1.06	× 1.20 × 1.40	÷ 1.04	54.37 0.32	1.42 0.86	× 2.06 ÷ 4.14	× 1.60 × 1.00	× 8.53
Kyleigh	C I	18.92 14.43		1.16 1.31	× 1.03 × 1.07	× 1.60 × 2.30	÷ 1.04	46.23 0.74	1.28 0.55	× 1.36 ÷ 1.36	× 1.50 × 2.70	× 1.85

*C* correct; *Cel.* celeration; *I* incorrect; *II* improvement index; *NT* notation type, *SD* standard deviation

During intervention, all three students showed consistency with performance. Alyssa, Jacob, and Kyleigh had correct notations-per-minute levels between 46 and 54, and 0 incorrect notations per minute. Jacob (× 2.06 and ÷ 4.14) and Kyleigh (× 1.36 and ÷ 1.36) had accelerating correct notations and decelerating incorrect notations. Alyssa also had accelerating correct notations (× 1.20) but slightly accelerating incorrect notations. Such a combination of scores resulted in improved II measures for Alyssa (× 1.12), Jacob (× 8.53), and Kyleigh (× 1.85), or, in each case, the quality of performance bettered during the intervention.

Table 2 displays between-condition metrics for both correct and incorrect notations for each student. Students showed immediate and lasting intervention effects. Alyssa (× 4.86), Jacob (× 1.88), and Kyleigh (× 1.30) all had jumps up, or immediate increases in level, from baseline to intervention for correct notations. Other than Alyssa, who remained at 0 for incorrect notations, Jacob and Kyleigh had tremendous jumps down, or immediate decreases in level, of ÷ 9.67 and ÷ 5.33 for incorrect notations. Comparing levels between conditions, all students had increasing level multipliers for correct notations ranging from × 2.44 (Kyleigh) to × 5.02 (Alyssa). Similarly, all students demonstrated decreasing level multipliers for incorrect notations, ranging from ÷ 83.34 (Jacob) to ÷ 2.69 (Alyssa).

**Table 2:** Between-condition measures of student notations

Student	NT	Frequency multiplier	Level multiplier	Bounce change	Celeration multiplier	II change
Alyssa	C I	× 4.86 × 1.00	× 5.02 ÷ 2.69	÷ 1.29 1.00	× 1.61 ÷ 1.32	× 2.11
Jacob	C I	× 1.88 ÷ 9.67	× 3.45 ÷ 83.34	× 1.33 ÷ 1.40	× 2.02 ÷ 3.39	× 8.87
Kyleigh	C I	× 1.30 ÷ 5.33	× 2.44 ÷ 19.50	÷ 1.07 × 1.17	× 1.32 ÷ 1.46	× 1.92

*C* correct; *Cel.* celeration; *I* incorrect; *NT* notation type

A few of the measured behaviors saw decreasing bounce change scores, suggesting a tightening of stimulus control due to the intervention. Bounce change measures for correct notations for Alyssa (÷ 1.29) and Kyleigh (÷ 1.07) and incorrect notations for Jacob (÷ 1.40) all showed decreases. Incorrect notations for Kyleigh (× 1.17) and correct notations for Jacob (× 1.33) did become more variable in the intervention. Unlike bounce change scores, all three students had consistent celeration multiplier scores for both measures and strong II change scores.

Following instruction, Alyssa (× 1.61), Jacob (× 2.02), and Kyleigh (× 1.32) had accelerating correct notations per minute as compared to baseline. As correct notations accelerated, incorrect notations turned down or decelerated for each student as well, ranging from ÷ 1.32 (Alyssa) to ÷ 3.39 (Jacob). Looking at the II change measures, which consider the change in quality from baseline to intervention, all three students showed dramatic improvement. Two of the three students (Alyssa and Kyleigh) showed approximately a 100% improvement in quality. Furthermore, Jacob’s × 8.87 II change measure demonstrated a 900% improvement in performance quality.

In the pre-think-alouds, both Jacob and Alyssa described their method of solving the problem as “*x* + 8 = 15.” Jacob described his process as asking himself what number added to “8” equaled “15.” Alyssa’s process involved similar trial and error. In her pre-think-aloud, she stated, “*8 minus 8 plus 15 minus 15. Minus 2 is 2 is 13 and then 13 and then 8 will become a 10 minus 15, 3. Wait. No, no*” before eventually arriving at the correct value of *x*. Kyleigh described it without much detail, stating, “*I minus the number,*” with no further explanation or detail.

After the intervention, all students increased the detail with which they described the process to the solution. Both Jacob and Alyssa described the steps in the task analysis. Jacob said, “I minus 7 because it is the inverse act,” then went on to demonstrate doing so on both sides. Alyssa said, “Minus 8, minus 8, *x* equals 7,” verbally noting each step. Kyleigh significantly increased her explanation, stating, “This becomes a minus” in reference to the addition sign, then adding, “You put 8 under... Then you also do it for the answer side...15 minus 8...which would be 7, and that’s the answer for *x*.”

The generalization probe contained eight problems with various presentations of single-step equations. Alyssa completed seven of eight problems correctly, with her error relating to computation. Jacob also completed seven of eight problems correctly. His error was related to the presentation of *x* with a fraction bar to represent division, as opposed to the traditional division sign (÷) used in the dependent variable assessment. Kyleigh completed all generalization problems correctly.

All three participants responded to the social validity questionnaire, which included closed- and open-ended questions. Closed-ended questions used a five-point Likert scale. Average student responses indicated the intervention did make meaningful change in their behavior related to the skill of solving one-step equations (*M* = 4.00, *Mdn* = 4.00, *SD* = 0). Open-ended responses indicated students felt these skills would be important for “real-life skills.” On closed-ended questions, students reported that the intervention resulted in them feeling significantly more confident in solving one-step equations (*M* = 4.67, *Mdn =* 5.00*, SD =* 0.58). Students felt slightly less strongly about the intervention being practical and time-efficient *(M* = 4.33, *Mdn* = 4, *SD =* 0.58). When asked whether they felt the intervention would be valuable in the context of their classroom, Jacob shared that only one of his teachers focused on speed, so he thought they might want to incorporate it. Both Alyssa and Kyleigh connected the intervention to problems they faced in their math class, with Alyssa explaining that “it helped with the thing we are working on in class.” Generally, students responded that they would recommend the intervention to other students (*M* = 5.0, *Mdn* = 5.0, *SD* = 0).

## Discussion

Promoting algebraic achievement supports students’ access and success in advanced mathematics courses (Fayer et al., 2017; Gaertner et al., 2014; Kim et al., 2015). Standardized assessment data suggest students with disabilities struggle to make meaningful growth in mathematics beyond eighth grade, which coincides with the appearance of mathematics containing compound, complex skills (Cortiella, 2011; Provasnik et al., 2016). As with other subjects such as reading, strong knowledge of and fluency related to foundational skills supports achievement in higher levels of mathematics (Lin & Powell, 2022; Powell et al., 2019). Therefore, the current study involved an intervention focused on improving students’ fluent responding with a foundational mathematical skill: the ability to solve one-step algebraic equations.

Overall results suggest strong experimental effects between the intervention and students’ correct and incorrect notations (Kazdin, 2011). On average, students went from attempting one to two problems correctly per minute and using inefficient strategies to solving over seven problems efficiently and accurately in one minute. Generalization probes suggest students used their knowledge to respond to varied presentations of single-step equations in which they were not explicitly trained in the intervention. Qualitative data from the pre- and post-think-alouds imply students changed their orientation to equations, moving from a trial-and-error approach to a more systematic use of algebraic procedures. Finally, social validity data indicated students would recommend the intervention to other students, citing its relevance to their classwork and increased confidence from consistent practice.

While previous equation-solving interventions represented in the literature are generally effective, the current intervention demonstrates student improvement in not only accuracy but also in speed, a measurement characteristic not often reported or graphed. Current research on algebraic equation-solving interventions also frequently used manipulatives or concrete representations to support concept knowledge (e.g., Bouck et al., 2019; Chapman et al., 2019; Jimenez et al., 2008; Long et al., 2020; Satsangi et al., 2016, 2018a, 2018b). While the use of manipulatives is impactful for conceptual development in initial instruction, the long-term use of manipulatives may inhibit student performance and become less feasible as algebraic procedures become more complex. Specifically, manipulatives may slow down student responding. Manipulatives may also create prompt dependency. Additionally, the current intervention achieved dramatic improvements using a shorter intervention duration (less than 10 minutes) across only 5–10 sessions.

The current results mirror findings from other research on effective frequency building and instruction for academic skills, such as words read (e.g., Lambe et al., 2015) and sentence writing (e.g., Datchuk et al., 2015). In addition to the direct benefit (i.e., more correct notations and fewer incorrect notations) demonstrated by the current study, all three students displayed positive side benefits, such as proper responding to a wide variety of single-step untrained equations. Again, students engaged in an effective, purposeful practice, and exhibited similar accurate responses to untrained situations (e.g., Kostewicz et al., 2020).

The positive results of the current study may be explained by the characteristics of frequency-building interventions within a precision teaching framework. In precision teaching, four actions completed in a cyclical nature guide decision-making: pinpointing observable and measurable behavior, recording via daily measurement, changing behavior through the iterative process of analysis and decision-making, and trying again by continuing to strive for positive learning outcomes (Kubina & Yurich, 2012). Within the design and delivery of the frequency-building intervention, we clearly defined or pinpointed each behavior using task analysis for the procedure of solving single-step equations. Each behavior had a consistent learning channel, defined by a specific combination of stimulus input and response output (e.g., see–say, hear–do; Kubina & Yurich, 2012). Specifically, students saw a written problem in a specific format and wrote in response to form a pinpoint of see notation/write notation. The current task analysis is different from prior literature, which often included steps that required attention to different modes of presentation. For example, prior interventions using manipulatives required students to both see the problem/write answers and see markers/touch markers (Jimenez et al., 2008). Because learning channels may be independent of each other, keeping response forms consistent across the task analysis via clearly pinpointed behaviors may have accelerated learning (Lindsley, 1996).

Prior to the intervention, as self-described in the pre-think-aloud, students prompted themselves via covert speech, showing little written work. Students also showed little written work on baseline probes despite prompts to do so. In the intervention, behavior was clearly defined, taught, and reinforced, students appeared to rely less on covert speech and guesswork. Even in nonroutine problems, strategies of “guess and check” have demonstrated little value in supporting students who struggle in mathematics (Arslan & Altun, 2007). Instead of engaging in guesswork, students aligned their verbal behavior to mirror the task analysis, supporting a more efficient and systematic approach to problem-solving.

The detail provided within student notations facilitated more precise feedback. Consider the problem *x* ÷ 3 = 9. If a student merely wrote *x* = 6, we would not know the precise nature of their error. The student could have correctly used division but erred in the computation; the student also could have erroneously used subtraction. However, when students write each step to their problem, the teacher no longer must guess at the nature of the error. Instead, the teacher can directly address the precise student error, allowing for more improved formative assessment and more efficient behavior change (Beesley et al., 2018). As mentioned, feedback related to computation errors or the task analysis steps. In the post-think-aloud, students more readily used language mirroring the task analysis in their explanation. The language may have been bolstered by the precise feedback.

The frequency-building intervention also emphasized the speed of student responses within both recording and changing the student behavior. Related to recording, the dependent variable measurement was not constrained by artificial ceilings, allowing for a more accurate understanding of the behavior's rate of change. The inclusion of time as a feature of measurement also supports a view of mastery extending beyond mere accuracy (Binder, 1996). Including fluent performance as a component of mastery supports the attainment of critical learning outcomes related to fluency, such as endurance, generalization, and application (Binder, 1996; Stocker et al., 2019). Given the sequential nature of mathematics, developing fluent performance on element skills such as solving equations can more readily support students’ using those skills flexibly as part of more complex problem-solving processes and compound skills (Spooner et al., 2019; Stocker et al., 2019).

## Limitations

Some limitations exist in the current study. Owing to school procedures, we could not obtain detailed characteristics of participants and thus relied primarily on our own screening and broad disability categories. Additional data may have provided a clearer picture of participants that could expand relevance. We also did not collect maintenance data, given external time constraints related to the school year. Maintenance data may have provided further insight into whether the specific performance standard of 60–80 correct notations per minute accurately captures fluent performance. We also used experimenter-created social validity measures, which were not delivered anonymously. Another limitation relates to the specific task analysis used to guide the measurement of correct and incorrect notations. We narrowly counted incorrect notations as those that did not follow a specific procedural format. Other procedures for solving equations with more or fewer notations may also yield positive and valid results. Furthermore, requiring notations after a student becomes fluent may be unnecessary, as they rely less on the notations as prompts.

Although procedural flexibility remains important to consider in problem-solving (Rittle-Johnson, 2017; Star & Newton, 2009), the current study’s primary research question did not aim to tackle the development of procedural flexibility but rather focused on speed and accuracy. While generalization data suggest students did generalize skill without being explicitly taught new procedures, these claims should be tempered. Finally, disruptions in the school schedule, including days when the school had standardized testing, resulted in the intervention sometimes being delivered at varied times of the day.

## Implications for Practice

The current study has critical implications for algebraic instructional practices for students with learning disabilities. First, when teaching skills, educators should pinpoint and clearly define behaviors in the procedures for solving equations (Kubina & Yurich, 2012). Then, teachers should consider how students can demonstrate those behaviors in a consistent learning channel (e.g., see–write). Having students overtly perform behaviors in a way that can be measured via “showing their work” can enhance the quality of feedback by allowing teachers to provide precise corrections versus making assumptions about covert behaviors.

Educators may consider how to incorporate timing into their interventions after the initial acquisition of a given skill. Students in the present study had some familiarity with equations. After even a brief exposure to a concept, the current study suggests that even a small dosage of 5–10 interventions across two to three weeks can support dramatic improvements in the speed and accuracy of students’ solving equations. The current intervention was delivered in a 1:1 format, which may be readily translated to existing tier 3 interventions in the context of a multitiered system of supports. With minor modifications (e.g., using peer tutors or worked examples), the intervention may be suitable for tier 2 or tier 1 implementation. Finally, given the broad success of frequency building across a variety of educational skills, mathematics educators may consider how to expand the practice beyond procedures for solving equations. Researchers have employed frequency building in pre-algebraic skills related to the order of operations (Stocker & Kubina, 2021). By considering clearly defined behaviors within algebraic procedures, educators can incorporate frequency building across a variety of topics to support student achievement.

## Implications for Research

The current study extends the study of algebraic interventions for students with disabilities. Furthermore, we extend the literature base of precision teaching and, more specifically, frequency building to applications beyond simple computations, such as addition, subtraction, and multiplication (Gist & Bulla, 2020). Given the sequential nature of procedural knowledge in mathematics, additional research should extend the current investigation to two-step and multistep equations. In addition to asking questions about the effects of frequency building on incorrect and correct notations, research may also be designed to answer questions about how fluent performance may relate to generalization and application to novel problem types and what dosage is most beneficial for students. Researchers may also consider varying features of practice, such as using an arbitrary number of timings instead of a contingency-based number of timings.

The graphical display of results in the current study provided unique advantages and should be used in future research. Instead of relying on traditional visual analysis, the current study used the SCC to guide both instructional decision-making and the evaluation of efficacy. The use of the SCC prevented the manipulation or inaccurate depiction of graphs. Graphs that do not adhere to graphical standards remain prevalent in special education single-case research and may bias the interpretation of the results (Kubina et al., 2021). Future use of the SCC in research could more readily allow for comparisons across studies, especially related to the development of students’ fluent performance.

Finally, perceptions of timing as being related to math anxiety may need to be further explored, understood, and countered to promote widespread uptake of frequency-building procedures. Researchers posit that mathematics anxiety influences performance and development of higher-level math skills (Dowker et al., 2016). Some researchers from non-behavior analytic standpoints associate mathematics anxiety with timed tests, arguing explicitly for the removal of timing in favor of methods that instead promote number sense (Boaler, 2015). Our social validity results indicated students felt more confident, and open-ended responses did not include expressions of timing and anxiety. The timing procedures in the present study were intentionally designed for students who demonstrated prerequisite skills. Explorations of mathematics anxiety grounded in behavior analytic frameworks should thus further explore student experiences with environmental variables related to timing procedures, such as task demands, criteria for reinforcement, and evaluation of a students’ learning history as relates to timing. Such evaluations could challenge the association between timing and anxiety. The alternative—removing timing without attention to environmental variables that may trigger anxiety or escape-maintained behaviors—may remove an opportunity for students to achieve fluent levels of performance, further exacerbating potential negative outcomes (VanDerHeyden & Codding, 2020).

## Conclusion

The present study demonstrated powerful, positive effects of a frequency-building intervention on three participants’ skills in relation to solving one-step equations quickly and accurately. The efficient and dramatic improvements provide evidence that frequency building can be successfully embedded into higher-level math tasks for students with disabilities. Data indicated students generalized their skills to varied presentations of one-step equations. Future research may continue to consider how to understand the relationship between frequency building and generalization or application. Expanding the work to other higher-level algebraic processes would also target a much-needed area of research.

## Data Availability

The datasets generated during and/or analyzed during the current study are available from the corresponding author upon reasonable request.
